# Could Homeopathy Become An Alternative Therapy In Dengue Fever? An example Of 10 Case Studies

**Published:** 2018

**Authors:** Seema Mahesh, Mallappa Mahesh, George Vithoulkas

**Affiliations:** * Centre for Classical Homeopathy, Vijayanagar, Bangalore, India; ** University of the Aegean, International Academy of Classical Homeopathy, Alonissos, Northern Sporades, Greece

**Keywords:** dengue fever, homeopathy, individual therapy

## Abstract

**Background:**Dengue fever is one of the most rampant epidemics in India of late and any therapy that may help limit the sickness and hospital admissions is worth considering. In India complementary and alternative medicine physicians are medically trained and hence have a role to play in delivery of public health.

**Case Series:**We present a retrospective case series of 10 Indian patients who were diagnosed with dengue fever and treated exclusively with homeopathic remedies at Bangalore, India.

This case series demonstrates with evidence of laboratory reports that even when the platelets dropped considerably there was good result without resorting to any other means.

**Conclusions:**A need for further, larger studies is indicated by this evidence, to precisely define the role of homeopathy in treating dengue fever. This study also emphasises the importance of individualised treatment during an epidemic for favourable results with homeopathy.

**Abbreviations:**DF: dengue fever, NS1: non-structural protein 1 antigen, IgG: immunoglobulin G, IgM: immunoglobulin M, +ve: positive, -ve: negative, WBC: white blood cells, RBC: red blood cells, ESR: erythrocyte sedimentation rate

## Background

Dengue is one of the most prevalent epidemics in India of late; increasing from 30,000 cases in 2010 to 100,000 in 2016 it has become a big concern to public healthcare [**1**]. Even though most cases recuperate with little medication, considering the dangerous potential haemorrhagic complications, it is essential to have a system in place that can efficiently meet this challenge. In a country where medical care in any form is many times difficult for the population to access, Complementary and Alternative physicians are welcome to lend a hand thus lightening the burden on manpower requirement and economy.

Homeopathy has been proven to be effective as prophylactic in large populations of endemic dengue areas [**2**] but to the best of our knowledge this case series is the first of its kind providing treatment of individual case details with the corresponding laboratory reports.

## Case Series

(**Tables 1**, **2**, **3**, **4**, **5**, **6**, **7**, **8**, **9**, **10**: Cases description)

The 10 patients were treated for DF at the Centre for Classical Homeopathy, Bangalore, India. In certain cases, patients resorted to antipyretics in the beginning at the onset of fever, but once the diagnosis of dengue was made, all patients were treated only with homeopathy.

The diagnosis was usually made on the first, second or third day after the onset of fever with a blood test to check for the presence of non-structural protein 1 antigen (NS1), immunoglobulin M (IgM) and immunoglobulin G (IgG). NS1 is evident in the blood at the early stages of infection and indicates the presence of an infection. IgM and IgG are detectable considerably later [**3**]. In these cases, the presence of NS1 was taken as the indication to begin dengue-targeted treatment, and the stability in terms of symptoms and blood parameters (mainly platelets) was considered the indicator to stop treatment. A complete blood count was performed at the beginning and any abnormal parameters (Platelets, WBCs or NS1) were monitored regularly until they stabilised (**Tables 1**, **2**, **3**, **4**, **5**, **6**, **7**, **8**, **9**, **10**)

**Table 1:** Case 1: 20 Year old male patient on treatment for psoriasis chronically

Date	Blood test results	Signs and symptoms	Homeopathic Remedy
19/06/2015	Hb: 15.8 g% WBC: 3,500 cells/cu mm N: 62; L: 33; E: 4; M: 1; B: 0; ESR: 5 mm/hour RBC: 5.58 million/ cu mm; Platelets: 1,00,000/cu mm Dengue NS1: -ve IgG: +ve Ig M: -ve		Patient did not consult till 2 days later
20/06/2015	Hb: 14.5 g% WBC: 2,900 cells/cu mm N: 56; L: 49; E: 2; M: 3; B: 0; ESR: 10 mm/hour RBC: 5.71 million/ cu mm; Platelets: 80,000/cu mm Dengue NS1: -ve IgG: +ve Ig M: -ve		
21/06/2015	Platelets: 70,000/cu mm	Fever since 3 days Craves Buttermilk	Thlaspi bursa pastoris 200C
23/06/2015	Platelets: 1,50,000/cu mm		Stop treatment
26/06/2015	Platelets: 2,70,000/cu mm		

**Table 2:** Case 2: 47 Year old female patient on treatment for hypothyroidism chronically

Date	Blood test results	Signs and symptoms	Homeopathic Remedy
27/06/2016	Hb: 12 g% WBC: 4,200 cells/cu mm N: 76.4; L: 14; E: 0.4; M: 8.6; B: 0.6; ESR: 25 mm/hour RBC: 4.18 million/ cu mm; Platelets: 1,70,000/cu mm Dengue NS1: +ve IgG: -ve Ig M: -ve	Severe body ache with fever, nausea on seeing food, abdominal distension - cannot lie on her back has to lie to the right; cannot tolerate any food can drink tea and warm water Few Bleeding spots were apparent on her oral mucosa	Patient did not consult till 2 days later
29/06/2016	Platelets at 10.40am: 17,000 cells/cu mm at 1.50 pm: 18,000 cells/cu mm at 7.00pm 14,000 cells/cu mm	She was excessively tired and weak. was hospitalised for expected emergency but no treatment was given	Lycoodium 200C
30/06/2016	Platelets at 5 am: 23,000 cells/cu mm at 4.30 pm: 42,000 cells/cu mm	She was generally feeling fine and was able to eat; She was discharged from the hospital	No change
01/07/2016	Platelets: 1,28,000 cells/cu mm	Feeling well	Treatment stopped
03/06/2016	Platelets: 2,08,000 cells/cu mm		

**Table 3:** Case 3: 28 year old female patient on treatment for bronchial asthma and hypothyroidism chronically

Date	Blood test results	Signs and symptoms	Homeopathic Remedy
14/09/2016	Hb: 13.1 g% WBC: 4,100 cells/cu mm N: 69.4; L: 24; E: 6; M: 1; B: 0; ESR: 13 mm/hour RBC: 4.36 million/ cu mm; Platelets: 62,000/cu mm Dengue NS1: +ve IgG: -ve Ig M: -ve	Severe body ache and tiredness with fever - the family admitted her to a hospital	She was at hospital the first 2 days so did not take homeopathy
15/09/2016	Platelets: 52,000 cells/cu mm	Patient was worse than the previous day in general	
16/09/2016	Platelets: 30,000 cells/cu mm	She exhibited oral mucosal hemorrhagic spots She could only drink lemonade and nothing else	Ptelea 200C
17/09/2016	Platelets 25,000 cells/cu mm	Genrally her condition was better than previous day - she could eat a little but still too weak	No change
18/09/2016	Platelets: 45,000 cells/cu mm	Patient felt fine and went home from the hospital	No change
19/09/2016	Platelets: 80,000 cells/cu mm	Feeling well	Treatment stopped
21/09/2016	Platelets 2,50,000 cells/cu mm		

**Table 4:** Case 4: 32 year old male patient on treatment for depression chronically

Date	Blood test results	Signs and symptoms	Homeopathic Remedy
30/05/2017	Dengue NS1: +ve IgG: -ve Ig M: -ve platelets: 1,40,000 cells/cu mm WBC: 6,800 cells/cu mm	Headache and pain in sacrum with fever; nausea at the sight of food; craving for refreshing juice	Acidum phosphoricum 200C
02/06/2017	Dengue NS1: -ve IgG: -ve Ig M: -ve Platelets: 1,41,000	Feeling well	Treatment stopped

**Table 5:** Case 5: 34 year old male not on any treatment before now

Date	Blood test results	Signs and symptoms	Homeopathic Remedy
08/06/2017	Dengue NS1: +ve IgG: -ve Ig M: -ve Platelets: 81,000	Diarrhoea - exhausting along with fever; Was afraid to stay alone - always wanted someone with him; There were hemorrhagic spots on the oral mucosa	Arsenicum album 200C
09/06/2017	Platelets: 65,000	Patient was not better and was brought again to clinic. On examination the pulse was very slow in correlation to the temperature	Pyrogenum 200C
10/06/2017	Platelets: 75,000	Diarrhoea stopped; patient feeling better	No change
11/06/2017	Platelets 1,15,000	Feeling fine	Treatment stopped

**Table 6:** Case 6: 8 year old girl on treatment for recurrent acute infections from time to time

Date	Blood test results	Signs and symptoms	Homeopathic Remedy
15/06/2017	Hb: 12.4 g% WBC: 3660 cells/cu mm N: 61.8; L: 29.5; E: 0.1; M: 8.2; B: 0.4; RBC: 5.11million/ cu mm; Platelets: 2,32,000/cu mm Dengue NS1: +ve	High fever; Pulse slow in correlation to temperature Pain in extremities; Nausea at the smell of food	Pyrogenum 200C
16/06/2017	Hb: 12.6 g% WBC: 3170 cells/cu mm N: 30.9 L: 56.3; E:0.4; M: 10.7; B: 1.7; RBC: 5.21million/ cu mm; Platelets: 1,99,000/cu mm	Generally girl is well no complaints	No change
19/06/2017	Hb: 12.7 g% WBC: 5860 cells/cu mm N: 19; L: 72.7; E: 3.5; M: 4.3; B: 0.5; RBC: 5.26 million/ cu mm; Platelets: 2,39,000/cu mm Dengue NS1: -ve	Feeling well	Treatment stopped

**Table 7:** Case 7: 16 year old male patient on treatment for premature greying of hair since 3 months

Date	Blood test results	Signs and symptoms	Homeopathic Remedy
04/07/2017	Hb: 15.3 g% WBC: 6000 cells/cu mm N: 77; L: 20.7; E: 2; M: 1 RBC: 5.10 million/ cu mm; Platelets: 2,31,000/cu mm Dengue NS1: +ve IgM: -ve; IgG: -ve	Rise of temperature in the afternoon; Wants to drink warm water; decreased appetite	Lycopodium 200C
06/07/2017	Hb: 15.7 g% WBC: 3600 cells/cu mm N: 55; L: 34.7; E: 6; M 5 RBC: 5.23 million/ cu mm; Platelets: 2,11,000/cu mm Dengue NS1: -ve	Appetite better	No change
07/07/2017	Hb: 15.9 g% WBC: 2600 cells/cu mm N: 19; L: 72.7; E: 3.5; M: 4.3; B: 0.5; RBC: 5.27 million/ cu mm; Platelets: 1,86,000/cu mm Dengue NS1: +ve IgM: weakly positive; IgG: -ve	Generally well	No change
08/07/2017	Dengue NS1: -ve IgM: -ve; IgG: -ve		Treatment stopped

**Table 8:** Case 8: 41 year old female patient on treatment for hypothyroidism and polycystic ovarian syndrome

Date	Blood test results	Signs and symptoms	Homeopathic Remedy
24/07/2017	Hb: 11.2 g% WBC: 6900 cells/cu mm N: 48; L: 32; E: 12; M: 8; RBC: 4.97 million/ cu mm; Platelets: 2,16,000/cu mm Dengue NS1: -ve IgM: weakly +ve; IgG: -ve	Diarrhoea with fever; Pulse slow in correlation to the temperature	Pyrogenum 200C
28/07/2017	Hb: 10.5 g% WBC: 4200 cells/cu mm N: 60; L: 32; E: 4; M: 4; RBC: 4.70 million/ cu mm; Platelets: 4,33,000/cu mm Dengue NS1: -ve IgM: +ve; IgG: -ve	Diarrhoea reduced.	No change
05/08/2017	Hb: 10.1 g% WBC: 8200 cells/cu mm N: 65; L: 30; E: 3; M: 2; RBC: 4.46 million/ cu mm; Platelets: 7,91,000/cu mm Dengue NS1: -ve IgM: -ve; IgG: -ve	Feeling well	Treatment stopped

**Table 9:** Case 9: 2 year old child on treatment for delayed milestones

Date	Blood test results	Signs and symptoms	Homeopathic Remedy
29/07/2017	Hb: 12 g% WBC: 3120 cells/cu mm N: 26.8; L: 67.6; E: 0.4; M: 4.8; RBC: 4.9 million/ cu mm; Platelets: 1,42,000/cu mm Dengue NS1: +ve	Child was asymptomatic except for fever	No remedy administered
30/07/2017	Hb: 12 g% WBC: 3080 cells/cu mm N: 17; L: 76.1; E: 1.3; M: 5; RBC: 4.88million/ cu mm; Platelets: 1,09,000/cu mm	No symptoms	Arum triphyllum 200C (patient had been on the same remedy previously for his chronic complaint and in the absence of any acute symptomatology the same was repeated - a homeopathic therapeutic law)
31/07/2017	Hb: 11.7 g% WBC: 4290 cells/cu mm N: 21.6 L: 62.7; E: 5.5; M: 8.7;B: 1.5 RBC: 4.78 million/ cu mm; Platelets: 1,00,000/cu mm	No symptoms	No change
02/08/2017	Hb: 12.3 g% WBC: 5320 cells/cu mm N: 26.4; L: 61.7; E: 4.4; M: 6.8; B: 0.7 RBC: 4.99 million/ cu mm; Platelets: 1,47,000/cu mm; Dengue NS1: +ve	No symptoms	No change
05/08/2017	Dengue NS1: -ve		Treatment stopped

**Table 10:** Case 10: 21 year old female not on any treatment before now

Date	Blood test results	Signs and symptoms	Homeopathic Remedy
22/08/2017	Hb: 13.1 g% WBC: 3960 cells/cu mm N:30.2 ; L: 55.7; E: 2.5; M: 10.1; B: 1.5 RBC: 4.61 million/ cu mm; Platelets: 1,96,000/cu mm; Dengue NS1: +ve	High fever; Craving for tomato soup	Ferrum metallicum 200C
23/08/2017	Hb: 11.9 g% WBC: 5760 cells/cu mm N: 21.7; L: 66.3; E: 4.3; M: 6.8; B: 0.9 RBC: 4.23 million/ cu mm; Platelets: 1,95,000/cu mm; Dengue NS1: -ve	Temperature is normal and patient generally well	Treatment stopped

Normal reference range for blood parameters:WBC count: 4,000 to 11,000 cells/cu mm, Neutrophils (N): 40 - 75%, Lymphocytes (L): 20 - 40%,Eosinophils (E): 0 - 6%, Monocytes (M): 2 - 10%, Basophils (B): 0 - 2%, RBC Count: 3.8 - 4.8 million cells/cu mm, Haemoglobin: 11.5 - 15.5 g%, Erythrocyte Sedimentation Rate (ESR): 0 - 20 mm/hr, Platelets count: 1,40,000 to 4,50,000/ cu mm, NS1 (Non specific antigen1) : negative (-ve), Immunoglobulin G (Ig G) : negative (-ve), Immunoglobulin M (Ig M): negative (-ve)

## Results

Generally, DF patients with NS1 positivity go on to become seropositive for IgM by day 5 and demonstrate detectability of all three markers by days 5 – 6 [**3**]. The 10 cases in this report included 5 males and 5 females. The average time from the detection of NS1 until it became negative was 4.4 days (minimum of 3 days and maximum of 8 days). Five patients exhibited a decrease in platelets, which became normal with treatment. Nine patients were +ve for NS1 at the time of diagnosis, and 1 patient was IgG +ve (probably due to late diagnosis). In case 8, NS1 was +ve at diagnosis but became IgM + ve by day 4, and one week after receiving the homeopathic remedy, these returned to normal. However, this patient took 8 days to recover. Case 5 required two remedies in succession because the first one was unsuccessful, leading to further decrease in the number of platelets. The second remedy was successful, and the platelet count immediately increased. Though there were at least three cases demonstrating mucosal bleeding, none of these cases proceeded to shock or severe haemorrhage. The significance of the homeopathic treatment is that all patients maintained at least a fairly good general condition during the infection and were able to return to normal functioning in a short length of time. There was no evidence of any post viral syndrome which are common in these cases [**4**].

Most of these patients were already on homeopathic treatment for their chronic complaints, so they did not delay in approaching the homeopath. If instead there was considerable time lost between onset of dengue and seeking homeopathy, we cannot say for sure if such favourable results would have been achieved.

## Discussion

This case series is significant because in all cases, the prescription was based on the principles of classical homeopathy, which considers the individual signs and symptoms of every patient for remedy selection. Often, these signs and symptoms do not have any relation to the pathological process occurring in the individual; rather, they are considered to be part of the immune response to the pathological agent, which is an attempt to re-establish homeostasis. Therefore even in epidemics, where the pathogen and pathology are similar in all cases, each individual’s reaction to them is different [**5**]. We observe in this study that only 3 out of 10 cases required the same remedy (as they had very similar symptoms).

The remedies are derived from animal, plant and mineral kingdom. They are prepared by a special process called ‘potentisation’ which renders even the most toxic substances safe for use as medicines [**6**].

## Conclusion

This case series demonstrates that classical homeopathy has the potential to help treat dengue infection. Further larger studies are required to confirm the extent to which it may be employed. This study further demonstrates that it is essential to consider the individual symptoms, even in epidemics, to achieve favourable results from homeopathy.

## Take home lessons

Dengue is a real public health threat in India. Complementary and Alternative Medicine physicians may help lessen the burden as they are medically trained in this country.

Case series of 10 cases to demonstrate that individualised therapy with homeopathy for dengue yields favourable results.

It was possible to maintain even the dangerously low platelet situations without hospitalisation or cumbersome procedures.

None of the cases progressed to a post dengue syndrome which will be worth investigating as a potential benefit of homeopathic therapy

Further larger studies on the feasibility and the extent to which individualised homeopathy may be employed in dengue affected areas need to be conducted.

**Funding:**None.

**Conflict of interest:**Authors declare there are none

**Consent for publication:**A written consent for publication has been obtained from the patients

**Trial Registration and Ethical Committee approval:**Not applicable

**Acknowledgements**The authors acknowledge the help of patients and their kin in giving consent for publication of the details of their dengue episode and its treatment.
